# Tauroursodeoxycholic Acid (TUDCA) Relieves Streptozotocin (STZ)-Induced Diabetic Rat Model via Modulation of Lipotoxicity, Oxidative Stress, Inflammation, and Apoptosis

**DOI:** 10.3390/ijms25136922

**Published:** 2024-06-25

**Authors:** Nema A. Mohamed, Mohammed T. Ithmil, Ayman I. Elkady, Sherine Abdel Salam

**Affiliations:** 1 Department of Zoology, Faculty of Science, Alexandria University, Alexandria 21511, Egypt; nema.abdelhamed@alexu.edu.eg (N.A.M.); lab.mohammed1968@gmail.com (M.T.I.); ayman.elkady@alexu.edu.eg (A.I.E.); 2 Department of Biology, Faculty of Science, Al-Mustansiriya University, Baghdad P.O. Box 14022, Iraq; 3 Department of Biological Sciences, Faculty of Science, King Faisal University, P.O. Box 380, Al-Ahsa 31982, Saudi Arabia

**Keywords:** TUDCA, antidiabetic, hypolipidemic, antioxidant, anti-inflammatory, anti-apoptotic

## Abstract

Tauroursodeoxycholic acid (TUDCA) is approved for the treatment of liver diseases. However, the antihyperglycemic effects/mechanisms of TUDCA are still less clear. The present study aimed to evaluate the antidiabetic action of TUDCA in streptozotocin (STZ)-induced type 2 diabetes mellitus (T2DM) in rats. Fifteen adult Wistar albino male rats were randomly divided into three groups (n = five in each): control, diabetic (STZ), and STZ+TUDCA. The results showed that TUDCA treatment significantly reduced blood glucose, HbA1c%, and HOMA-IR as well as elevated the insulin levels in diabetic rats. TUDCA therapy increased the incretin GLP-1 concentrations, decreased serum ceramide synthase (CS), improved the serum lipid profile, and restored the glycogen content in the liver and skeletal muscles. Furthermore, serum inflammatory parameters (such as TNF-α, IL-6, IL-1ß, and PGE-2) were substantially reduced with TUDCA treatment. In the pancreas, STZ+TUDCA-treated rats underwent an obvious enhancement of enzymatic (CAT and SOD) and non-enzymatic (GSH) antioxidant defense systems and a marked decrease in markers of the lipid peroxidation rate (MDA) and nitrosative stress (NO) compared to STZ-alone. At the molecular level, TUDCA decreased the pancreatic mRNA levels of iNOS and apoptotic-related factors (p53 and caspase-3). In conclusion, TUDCA may be useful for diabetes management and could be able to counteract diabetic disorders via anti-hyperlipidemic, antioxidant, anti-inflammatory, and anti-apoptotic actions.

## 1. Introduction

Type 2 diabetes mellitus (T2DM) is a complex endocrine and metabolic disorder, which is characterized by deficient insulin secretion, insulin resistance (IR), and/or pancreatic islet β-cell failure to maintain normoglycemia [1]. In particular, T2DM is becoming a global challenge that affects millions of people of all ages, sex, race, and ethnic groups [2]. T2DM has been reported to be associated with serious complications, including retinopathy, nephropathy, liver damage, and cardiovascular diseases [3]. Evidence in the literature has indicated that under the T2DM condition, proinflammatory cytokines and chemokines increase, which also initiate oxidative stress through excessive production of reactive oxygen and/or nitrogen species (ROS/RNS) [4]. Furthermore, oxidative stress overload can compromise ß-cell function, trigger apoptotic cell death, and ultimately may lead to a progressive loss of ß-cell mass [5]. Therefore, the use of natural antioxidants as complementary or adjunct therapies can offer new avenues for the prevention and even treatment of T2DM. Tauroursodeoxycholic acid (TUDCA) is an endogenous bile acid derivative that has been documented to be clinically effective in the treatment of cholestatic liver disorders [6]. TUDCA has also been reported to improve inflammatory metabolic diseases such as atherosclerotic progression, persistent hyperglycemia associated with both types of diabetes, and kidney failure [7]. Furthermore, TUDCA has demonstrated anti-apoptotic properties in a number of neurological injuries [8,9]. Recently, TUDCA treatment was reported to reduce glucose homeostasis disturbance in streptozotocin (STZ)-induced Alzheimer’s disease pathology [10]. However, the precise mechanisms that regulate the potential antidiabetic effects of TUDCA remain elusive. Therefore, our objective was to assess the antidiabetic efficiency of TUDCA against STZ-induced T2DM in vivo while investigating the underlying biochemical/molecular mechanisms. Our results may have significant importance for the clinical translation of TUDCA as an adjunct therapeutic agent in the management of T2DM.

## 2. Results

### 2.1. TUDCA Improves Hyperglycemia in Diabetic Rats

**Figure 1:**
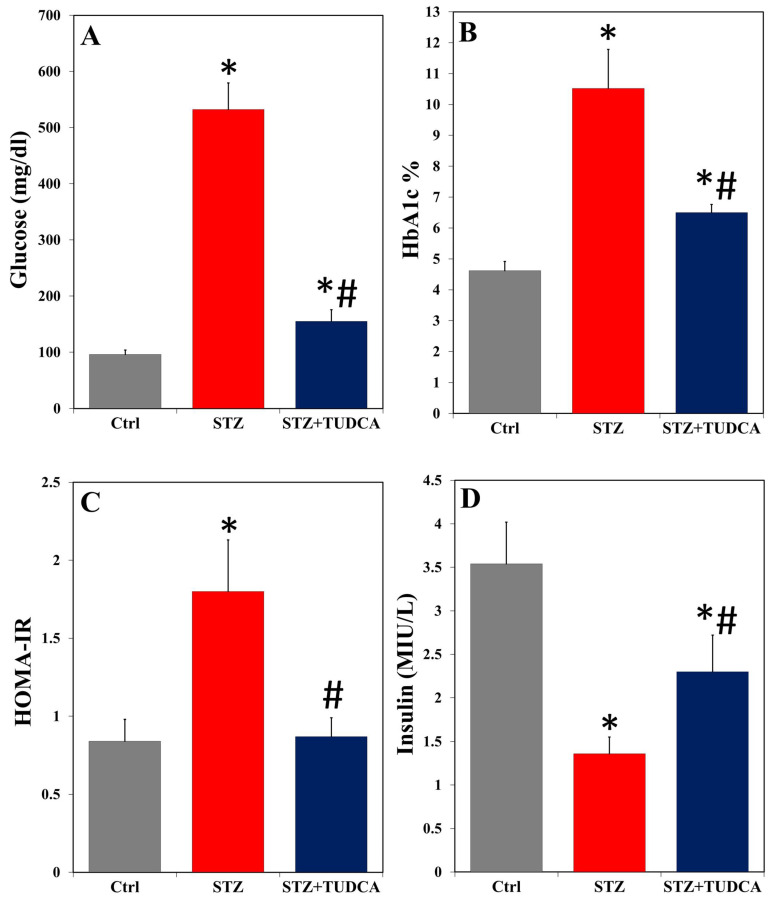
TUDCA decreased the levels of serum glucose (**A**), HbA1c% (**B**), and HOMA-IR (**C**) and increased insulin (**D**) in STZ-induced diabetic rats. Data are expressed as mean ± SD (n = 5). * *p* < 0.05 versus control (Ctrl), and # *p* < 0.05 versus STZ.

**Figure 2:**
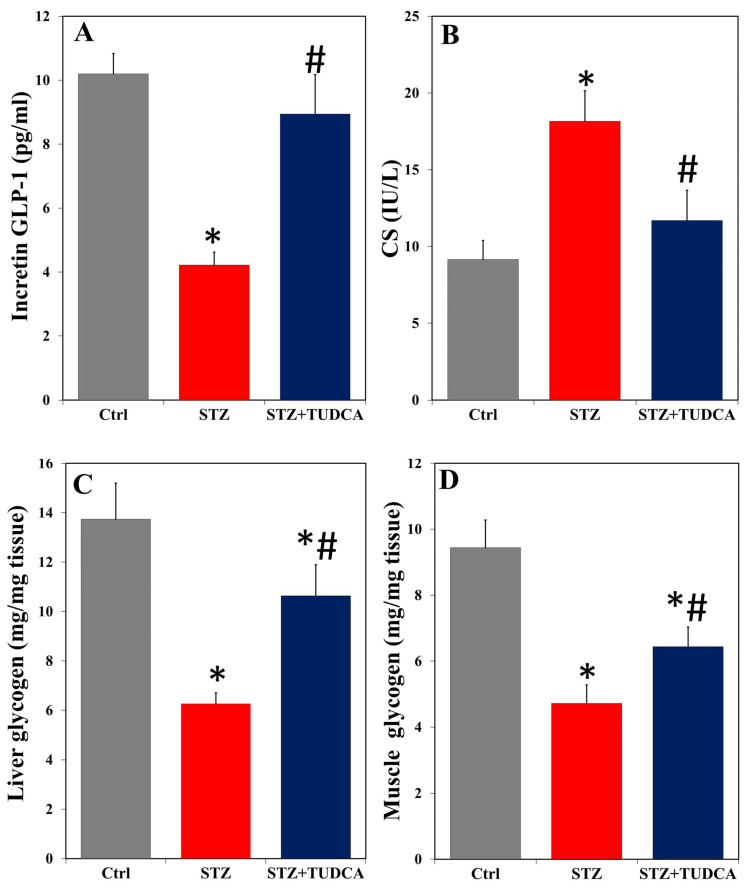
TUDCA increased serum incretin GLP-1 (**A**), decreased serum CS (**B**), and enhanced liver/muscle glycogen storage (**C**,**D**) in STZ-induced diabetic rats. Data are expressed as mean ± SD (n = 5). * *p* < 0.05 versus control (Ctrl), and # *p* < 0.05 versus STZ.

As shown in Figure 1, TUDCA treatment elicited significantly higher blood glucose (Figure 1A), HbA1c% (Figure 1B), and HOMA-IR (Figure 1C) in STZ-induced diabetic rats compared to the control. Furthermore, there was a significant decrease in the insulin levels (Figure 2D) of diabetic rats compared to normal animals. However, all these values were reversed in STZ+TUDCA-treated rats compared to diabetic rats. The results indicated that the treatment of diabetic rats with TUDCA induced a marked decrease in glucose, HbA1c%, and HOMA-IR and increased insulin compared to the STZ group. In particular, TUDCA was effective in returning HOMA-IR to normalized control levels.

### 2.2. TUDCA Effects on Serum Incretin GLP-1, CS, and Liver/Muscle Glycogen in Diabetic Rats

Next, we found that incretin GLP-1 was lower in the serum of diabetic rats (Figure 2A), while CS was drastically higher (Figure 2B) than in the corresponding controls. Treatment with TUDCA in diabetic rats significantly increased the level of incretin GLP-1 and reduced the activity of CS compared to STZ-induced diabetic rats, with recovery to the control values. It was also noticed that the liver/muscle glycogen levels were in high decline (Figure 2C,D) in diabetic rats compared to normal animals. Treatment with TUDCA significantly increased glycogen in the liver and muscle of STZ+TUDCA-treated rats compared to the STZ-alone treated group, but the values remained below normal.

### 2.3. TUDCA Effects on Dyslipidemia in Diabetic Rats

**Figure 3:**
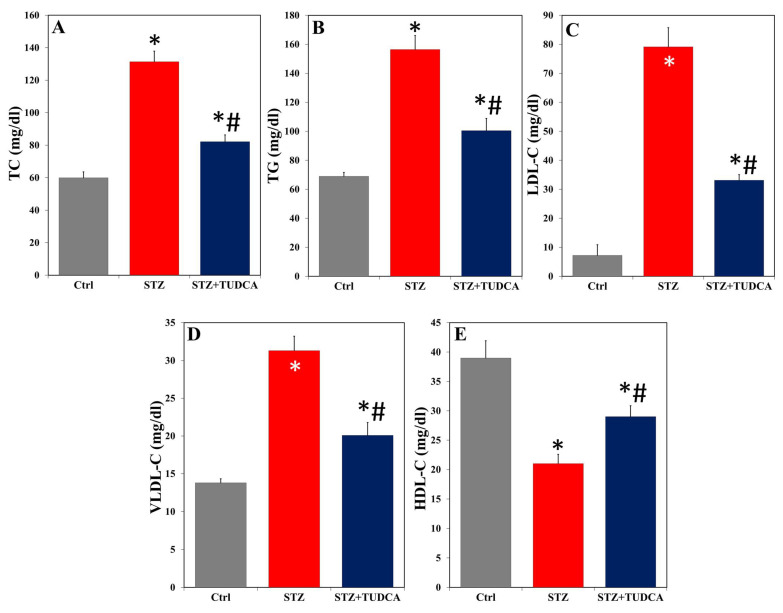
TUDCA decreased serum levels of TC (**A**), TG (**B**), LDL-C (**C**), and VLDL-C (**D**) and increased HDL-C (**E**) in STZ-induced diabetic rats. Data are expressed as mean ± SD (n = 5). * *p* < 0.05 versus control (Ctrl), and # *p* < 0.05 versus STZ.

The results obtained showed a statistically significant increase in the serum levels of TC (Figure 3A), TG (Figure 3B), LDL-C (Figure 3C), and VLDL-C (Figure 3D), while HDL-C significantly decreased (Figure 3E) in STZ-induced diabetic rats compared to the control values. Upon administration of TUDCA to STZ-treated rats, the levels of TC, TG, LDL-C, VLDL-C, and HDL-C were corrected relative to the STZ-alone group without reaching the normal levels.

### 2.4. TUDCA Effects on Proinflammatory Cytokines and PGE-2 in Diabetic Rats

**Figure 4:**
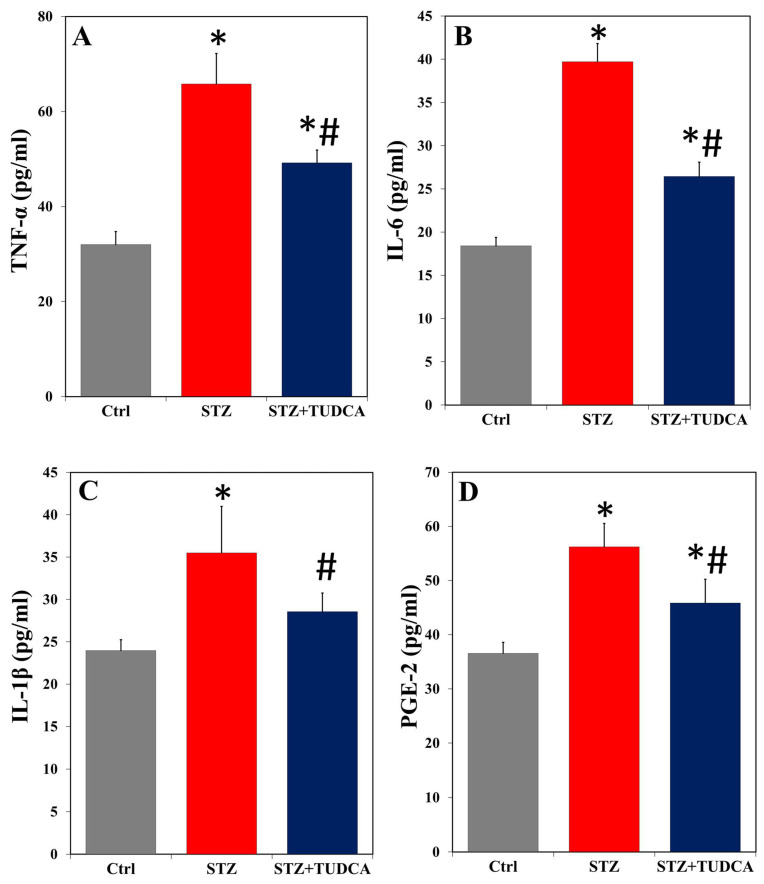
TUDCA decreased serum TNF-α (**A**), IL-6 (**B**), IL-1β (**C**), and PGE-2 (**D**) in STZ-induced diabetic rats. Data are expressed as mean ± SD (n = 5). * *p* < 0.05 versus control (Ctrl), and # *p* < 0.05 versus STZ.

Figure 4 depicts that there was a significant increase in the serum levels of proinflammatory TNF-α (Figure 4A), IL-6 (Figure 4B), IL-1β (Figure 4C), and PGE-2 (Figure 4D) in the diabetic group compared to the control. These key inflammatory factors significantly diminished in the STZ+TUDCA-treated rats compared to the STZ-induced diabetic rats. Specifically, TUDCA adequately reduced IL-1β down to the control values.

### 2.5. TUDCA Effects on Pancreatic Redox State in Diabetic Rats

**Figure 5:**
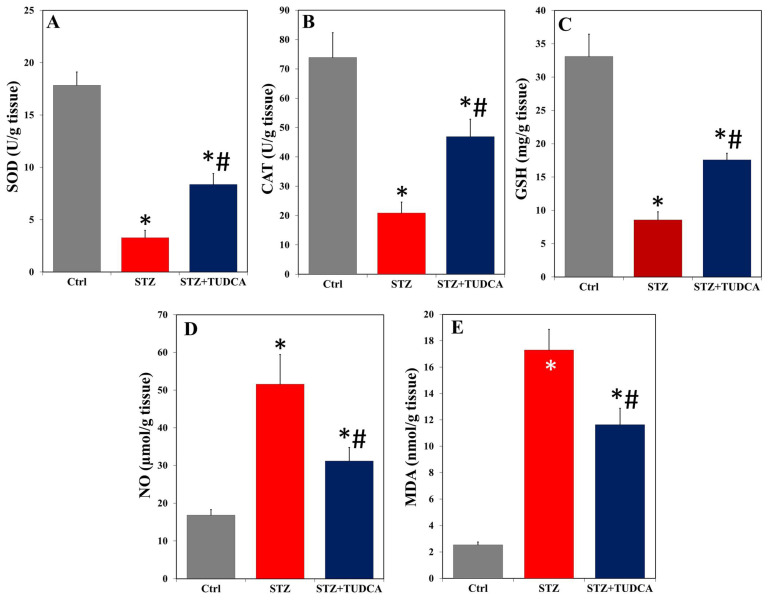
TUDCA increased pancreatic SOD (**A**), CAT (**B**), and GSH (**C**) and decreased NO (**D**) and MDA (**E**) in STZ-induced diabetic rats. Data are expressed as mean ± SD (n = 5). * *p* < 0.05 versus control (Ctrl), and # *p* < 0.05 versus STZ.

Our data showed that STZ treatment resulted in significant depletions in pancreatic SOD (Figure 5A), CAT (Figure 5B), and GSH (Figure 5C) compared to the control group. TUDCA treatment significantly counteracted these decreased levels in diabetic rats but not to the control baseline levels. However, there were significant elevations in the levels of NO (Figure 5D) and MDA (Figure 5E) in the pancreas of diabetic rats compared to the healthy control group. TUDCA significantly reduced NO and MDA in diabetic animals but could not bring them to normal values.

### 2.6. TUDCA Effects on mRNA Expression of iNOS, p53, and Caspase-3 in Diabetic Rats

**Figure 6:**
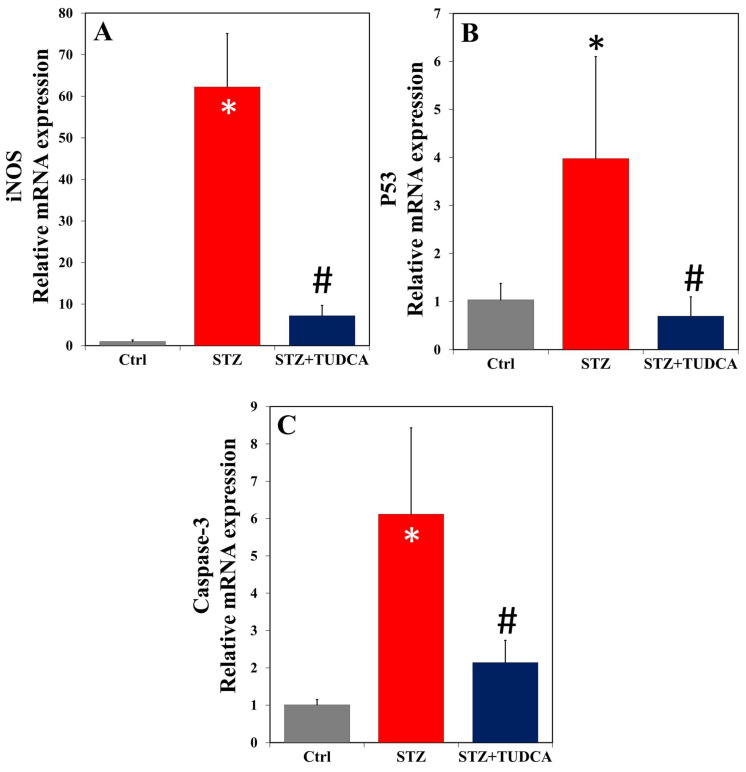
TUDCA downregulated pancreatic mRNA expression of iNOS (**A**), p53 (**B**), and caspase-3 (**C**) in STZ-induced diabetic rats. Data are expressed as mean ± SD (n = 4 or 5). * *p* < 0.05 versus control (Ctrl), and # *p* < 0.05 versus STZ.

The qRT-PCR results showed that the diabetic group (STZ) induced a substantial up-regulation of mRNA in pancreatic iNOS (Figure 6A), p53 (Figure 6B), and caspase-3 (Figure 6C) compared to the control. However, the administration of TUDCA as therapy in STZ-induced diabetic rats resulted in a marked downregulation of these genes compared to STZ-treated animals. Notably, in the STZ+TUDCA group, the expression of all the tested genes declined to the control levels.

## 3. Discussion

TUDCA and its taurine-free precursor UDCA have been used to treat human liver diseases, usually in long-term (chronic) oral doses of 10–20 mg/kg/day [11,12]. Furthermore, protection by UDCA/TUDCA extends beyond the liver to other organs and targets in the body. Preclinical data in nondiabetic models (e.g., [13,14,15,16,17,18]) showed that high ip/iv doses of UDCA/TUDCA (up to 500 mg/kg/day), administered as single or short-term injections, were safe and well-tolerated for therapeutic use. The aim of the current work is to assess the hypoglycemic action of TUDCA in STZ-induced diabetic rats. STZ has been extensively used as a drug of choice to induce diabetes in animal experiments [19]. It was reported that low ip doses of STZ (40–50 mg/kg b.w.) result in the destruction of some of the population of β-cells in the pancreatic islets, and residual β-cells remain, which produce insufficient insulin, causing the T2DM model [20,21]. The mechanism of the STZ-induced diabetic state includes its selective cytotoxicity to β-cells, which makes cells less active, leading to poor insulin sensitivity to glucose uptake by tissues and hyperglycemia [22]. Pancreatic β-cells are extremely vulnerable to oxidative damage by NO and free radicals due to the relatively low activity of antioxidant enzymes in the tissue [23]. The current results demonstrated that ip administration of TUDCA at a dose of 300 mg/kg b.w. has an antidiabetic effect by lowering the levels of glucose, HOMA-IR, and HbA1c% and improving the levels of insulin and glycogen in the liver and muscle. In a previous study, 20 days of treatment of TUDCA (500 mg.kg/day, i.p.) established normoglycemia and modulated insulin resistance in the liver, skeletal muscle, and adipose tissue of genetically obese ob/ob mice [24]. Comparatively, the lower ip dose of TUDCA used in this research was previously found to be helpful for the control of hyperinsulinemia in obese pre-diabetic mice [25]. Moreover, in silico studies predicted that TUDCA may also act as an insulin receptor agonist, which, in addition, can contribute to beneficial effects on insulin sensitivity [26]. In this context, TUDCA has emerged as an important candidate for T2DM; however, we still do not understand the complete picture of the mechanisms of action of this bile acid on glycemic control. Our data indicated a normalizing effect of TUDCA on the STZ-induced inactivation of serum incretin GLP-1, which further supports the antihyperglycemic properties of TUDCA. Incretin hormones (GLP-1 and GIP) have been reported to enhance insulin release from the pancreas and have been shown to exert proliferative and anti-apoptotic effects on the β-cells [27]. Previously, in vitro and in vivo reports have already pointed to bile acids as potent stimulators of GLP-1 secretion [28,29,30]. As already reported, the pancreas contains (static) quiescent ß-cells, which have the capacity to regenerate; in turn, the surviving cells multiply to replace the lost cells [31]. Therefore, it is possible that TUDCA was also responsible for the proliferation of ß-cells and the recovery of the STZ-induced lowered insulin level. There are already reports showing other natural ingredients that have regenerative potential for ß-cells [21,32].

Furthermore, our results are the first demonstration that TUDCA significantly reduced CS in sera from STZ-induced diabetic rats. Serum CS has been linked to atherosclerotic lesions and immune-inflammatory states in experimental animals [33]. Emerging evidence has implicated the accumulation of cell-permeable ceramide in pancreatic β-cells as contributing to β-cell destruction [34]. Early reports indicate that ceramide sphingolipid inhibits insulin-stimulated glucose uptake, insulin gene transcription, and glycogen synthesis [35,36]. Ceramide induces the complex signaling machinery required for the cascade activation of caspases [37]. In addition, ceramide has been shown to target the mitochondrial membrane, resulting in ROS/RNS production and the activation of cell apoptosis [38]. The inhibition of Akt by ceramide is also proposed in the mechanisms by which ceramide causes β-cell apoptosis [39]. On the basis of the previous background, it is plausible to speculate that the anti-apoptotic action of TUDCA may be mediated by counteracting ceramide production. These results are consistent with a previous in vitro observation by Liu et al. [40].

In the current study, we also detected hyperlipidemia in STZ-induced diabetic rats. The aberrant lipid profile was evidenced by elevated levels of TC, TG, LDL, and VLDL and decreased HDL. The robust production of ROS (pro-oxidants) is another outcome of hyperlipidemia under diabetic conditions [41]. The findings of our study revealed that TUDCA treatment effectively combated hyperlipidemia by lowering TC, TG, LDL-C, and VLDL-C and by raising HDL-C in diabetic rats. This suggests that TUDCA has a lipid-lowering action, which can be attributed to an improvement in insulin secretion and subsequent reduction in the synthesis of TC and TG. Our results are in agreement with the reported findings of Cui et al. [42], who used different concentrations of TUDCA (25/50/100 mg/kg b.w.) orally to treat high fat-diet (HFD)-induced hyperlipidemic mice.

Furthermore, we have investigated the effect of TUDCA on key mediators implicated in the downregulation of oxidative stress in STZ-induced diabetic rats. The STZ-induced prolonged hyperglycemia is associated with high levels of free radicals, which can lead to harmful effects such as (i) increased lipid peroxidation, (ii) altered antioxidant status, and (iii) impaired glucose metabolism in biological systems [43]. The imbalance between these pro-oxidant molecules and endogenous antioxidants (i.e., oxidative stress) plays an important role in insulin resistance pathogenesis [44,45]. Overproduction or under-removal of free radicals could overpower the body’s antioxidant mechanisms and develop several adverse effects commonly seen in T2DM, such as neuropathy, retinopathy, nephropathy, and vascular complications [46]. It is known that the SOD enzyme is regarded as the first line of defense against ROS-mediated cell disruptions by catalyzing superoxide radicals to molecular oxygen and peroxide [47]. However, CAT has a prime role in regulating the cellular level of hydrogen peroxide, and its catabolism protects the cells from oxidative assaults. In fact, CAT deficiency has been suggested to predispose to progressive pancreatic β-cell failure and diabetes [48]. GSH is a thiol-containing compound known to play a crucial role in scavenging hydroxyl radicals and singlet oxygen [49]. In our experiment, treatment with TUDCA reduced SOD, CAT, and GSH in the pancreas and restored the MDA level, suggesting a decrease in lipid peroxidation. Recent studies, consistent with our results, have reported that TUDCA can increase SOD activity and other antioxidants in isolated cardiomyocytes [50], retinal degeneration models [51], and spinal cord injury in mice [52]. Through the activation of Nrf2 antioxidant signaling in the human neuroblastoma SH-SY5Y cell line, TUDCA triggered the upregulation of antioxidant enzymes, which inhibit ROS accumulation and slow down destructive damage induced by oxidative stress [53].

Inflammatory conditions are associated with increased cellular levels of ROS/RNS and the production of proinflammatory molecules [54,55]. Thus, the anti-inflammatory effect of TUDCA may be related to its excellent free radical scavenging ability and antioxidant properties. Previous in vitro data obtained using cell cultures indicate that TUDCA suppressed proinflammatory stimuli induced by nitrite production [56]. Furthermore, following proinflammatory stimuli in glial cells, TUDCA has been reported to induce the transcriptional and translational inhibition of iNOS [57], whose expression is regulated by the NFκB pathway. Consistent with these studies, we have shown that TUDCA inhibited the downstream inflammatory cascade manifested by decreasing the mRNA expression of iNOS and the levels of TNF-α, IL-6, IL-1ß, and PGE-2 in diabetic rats.

Studies have strongly pointed to increased pancreatic β-cell apoptosis in diabetes mellitus, which occurs due to the involvement of oxidative stress and inflammation [58]. In the present study, the data confirmed the upregulation of proapoptotic genes such as p53 and caspase-3 in diabetic rats, which are in agreement with previously reported studies [59,60]. Following ROS insult, elevated levels of p53 promote Bax-induced cytochrome c release and stimulate the intrinsic (mitochondrial) apoptotic pathway, with subsequent activation of caspase-3, a key effector in the execution phase of apoptotic events [61]. On the other hand, high levels of NO can interact with superoxide anion to form a potent oxidant peroxynitrite, inducing apoptotic DNA fragmentation and p53-dependent apoptosis in pancreatic islets [62]. Interestingly, we found that TUDCA treatment decreased apoptosis in the pancreas, as seen with significant decreases in the mRNA expression of iNOS, p53, and caspase-3. Consistently, previous studies also reported the anti-apoptotic effects of TUDCA in apoptosis-related diseases [63,64]. In other injury models, UDCA, and its amidated conjugates, TUDCA and glycoursodeoxycholic acid, were shown to inhibit apoptosis by reducing ROS, inhibiting Bax translocation, and, consequently, cytochrome c release, with further blockage of caspase-3 (for review, see ref. [65]).

To further assess the in vivo antidiabetic action of TUDCA, we recommend examining histopathologic findings in the pancreatic islets. The lack of details regarding the time/dose-effect relationship of TUDCA on STZ-induced T2DM is among the limitations of this experiment, and future studies should adopt more TUDCA doses at different time intervals. In future, researchers should also investigate other molecular targets of TUDCA that may play a role in alleviating oxidative stress in the pancreas during experimental diabetes.

**Figure 7:**
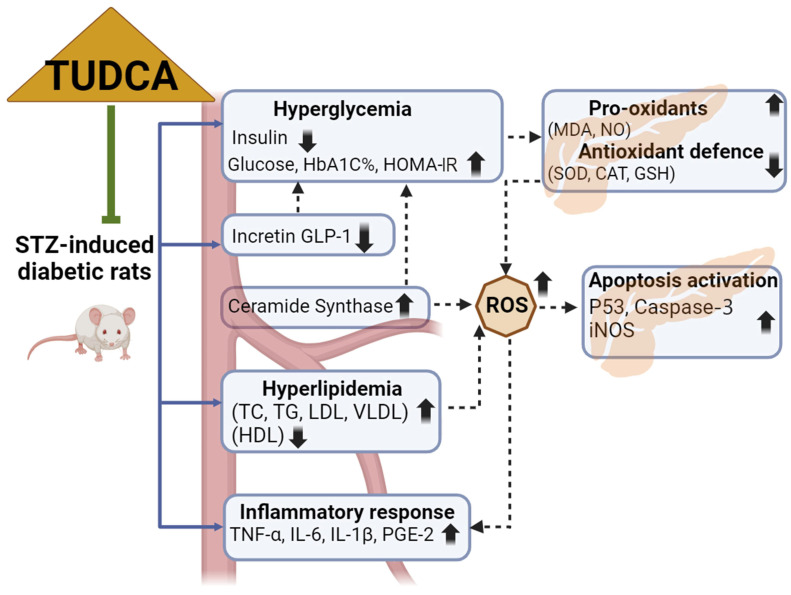
Schematic representation of the mechanism of action of TUDCA against the STZ-induced diabetic in vivo model. Created with BioRender.com.

Overall, this study introduces new information on the therapeutic role of TUDCA against T2DM (Figure 7). Based on our observations, the antihyperglycemic effects of TUDCA were accompanied by an increase in the levels of incretin GLP-1 and a decrease in CS activity. Treatment of diabetic rats with TUDCA could stabilize the intracellular redox status and prevent oxidative degradation in the pancreas. The anti-inflammatory effects of TUDCA may be related to the reduction in circulating proinflammatory proteins and cytokines and could result from boosting the antioxidant system and inhibiting iNOS-derived NO production. In addition, we provided experimental evidence that TUDCA possessed anti-apoptotic activity by downregulating the expression of P53 and caspase-3 in the diabetic pancreas. These findings therefore validate the claims that TUDCA is a promising intervention to control the diabetic milieu.

## 4. Materials and Methods

### 4.1. Chemicals

STZ was purchased from Sigma-Aldrich Chemical Co. (www.sigmaldrich.com), St. Louis, MO, USA. TUDCA was obtained commercially from Double Wood LCC, Philadelphia, PA, USA. All the other chemicals and reagents used in this study were of the highest analytical grade commercially available.

### 4.2. Animals

Adult male Wistar albino rats (180–200 g) were obtained from the animal breeding house at the Faculty of Agriculture, Alexandria University, Egypt. Upon delivery, the rats were kept at 22–25 °C, low relative humidity, and a 12 h light/dark cycle with standard balanced feed and tap water available ad libitum. They were accustomed to the lab conditions for 2 weeks prior to the study procedure. The animal experiments were approved by the animal care committee at Alexandria University (AU-IACUC Ref. No.: 04 22 02 12 1 02).

### 4.3. Experimental Design and Sample Collection

**Figure 8:**
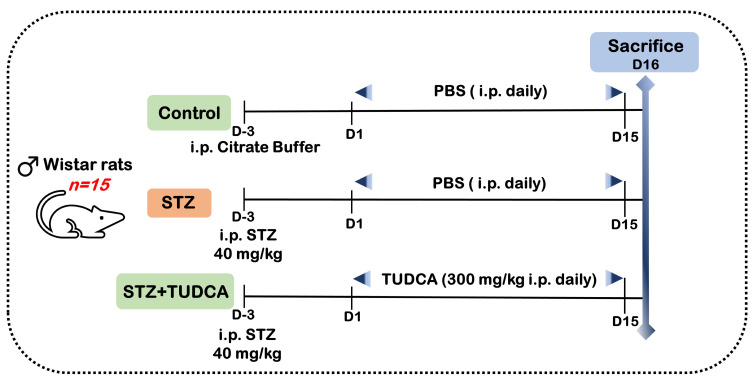
Timeline showing a summary of the study protocol.

The diabetic model was induced through a single i.p. injection of low-dose STZ (40 mg/kg b.w.), which was freshly prepared with sodium citrate buffer (0.1 M/L; pH 4.5) [66]. The STZ-injected animals received a glucose solution (20%) for 24 h to prevent STZ-induced initial hypoglycemia. A total of 72 h after STZ injection, tail blood was taken to determine the glucose level using a glucometer (Frankenberg, Germany) and reagent strips (Accu-Chek, Roche, Basel, Switzerland). Rats with a fasting blood glucose level of more than 200 mg/dL were selected for further experimentation. The rats were randomly divided into three groups (with five rats in each group), as follows: nondiabetic control (Ctrl), STZ (diabetic Ctrl), and STZ+TUDCA. TUDCA treatments (300 mg/kg b.w. dissolved in sterile PBS) were started after the induction of diabetes, and this was continued for 15 days [25]. Meanwhile, the rats in the control group received the same volume of vehicle(s) as the STZ- and STZ+TUDCA-treated animals (Figure 8). At the end of the experiment, the rats were deprived of food overnight and sacrificed after light ether anesthesia. The blood was allowed to clot in a centrifuge tube and the sera were centrifuged at 3000× *g* (Hettich Zentrifugen, Universal 32 R, Darmstadt, Germany) for 5 min at 4 °C. The serum was separated and stored at −20 °C pending biochemical analyses. Organs (liver, muscle, and pancreas) were obtained and prepared for further assays. The remaining parts of the pancreatic tissues were immediately submerged in liquid nitrogen and kept at −80 °C for molecular testing (qRT-PCR).

### 4.4. Evaluation of Serum Glycemic Markers

Blood glucose was determined using an enzymatic colorimetric method according to Trinder [67]. Insulin was measured using an immunosorbent assay kit (The Thermo Scientific™ Pierce™, Waltham, MA, USA) with the intra-assay coefficient of variation percent (CV%) < 10% and the inter-assay CV% < 12%. Insulin resistance (IR) was determined by calculating the homeostatic model assessment (HOMA)-IR index, as follows: HOMA-IR = [fasting blood glucose (mg/dL) × fasting insulin (mU/mL)]/405 [68]. HbA1c% was estimated using the fast ion-exchange resin separation method [69].

### 4.5. Assays for Incretin GLP-1 Level and CS Activity in Serum and Glycogen Quantification in Liver and Muscle

The serum incretin GLP-1 was assessed using the Mouse/Rat Glucagon-Like Peptide-1 (GLP-1 Active) ELISA Kit (Cat. Number: RSHAKMGP-011R, Shibayagi Co., Ltd., Gunma, Japan) according to the manufacturer’s instructions, with the intra-assay and inter-assay precision CV% < 5%. Rat CS activity was determined using an ELISA Kit (Cat. Number: SL1542Ra, SunLong Biotech Co., Ltd., Hangzhou, China). The intra-assay CV% for this test, as reported by the manufacturer, was <10% and the inter-assay CV% was <12%. The liver and muscle glycogen content was determined using the method of Huijing [70].

### 4.6. Quantitative Evaluation of Serum Lipids

The levels of TC, TG, and HDL-C were measured in the serum samples using the methods described, respectively, by Allain [71], Bucolo and David [72], and Lopez-Virella et al. [73]. The VLDL-C and LDL-C concentrations were calculated using Friedewald formulas: VLDL-C (mg/dL) = TG/5 and LDL-C (mg/dL) = TC − (HDL-C + VLDL-C) [74].

### 4.7. Detection of Serum Inflammatory Response

The serum levels of TNF-α, IL-6, IL-1β, and PGE-2 were measured using commercially available ELISA kits [TNF-α: Cat. Number: RK00029, Abclonal Co., Woburn, MA, USA; IL-6: Cat. Number: MBS726707, MyBioSource; IL-1β: Cat. Number: SEA563Ca, Cloud Clone Corp.; PGE-2: Cat. Code: RTFI01386, Assay Genie Co., Tokyo, Japan]. The intra-assay and inter-assay CV% were <10% and <15% for TNF-α, 7.2–7.8% and 5.5–6.3% for IL-6, <10% and <12% for IL-1β, and <8% and <10% for PGE-2, respectively, according to the corresponding manufacturer’s protocol.

### 4.8. Estimation of Oxidative Stress Biomarkers in the Pancreas

The tissues were removed, weighed, and washed in ice-cold normal saline, followed by homogenization in 2 mL phosphate buffered saline (*w*/*v*: 500 mg of tissue with 4 mL of PBS, pH: 7.4). The homogenates were centrifuged at 10,000× *g* for 20 min at 4 °C. The supernatant was collected in clean tubes and stored at −20 °C until the assays were performed. The tissue SOD activity was estimated using the method described by Nishikimi et al. [75], while the enzymatic activity of CAT was evaluated as described by Aebi [76]. The GSH level was determined according to the method of Beutler et al. [77]. The tissue NO was colorimetrically detected as nitrite according to the Griess reaction [78]. The lipid peroxidation index (MDA) was measured by monitoring the formation of reactive thiobarbituric acid substances [79].

### 4.9. Quantitative Real-Time PCR (qRT-PCR) Assay

Homogenization of 50–500 mg pancreas tissues in 1 mL of Lysis buffer (easy-BLUETM reagent) (iNtRON Biotechnology, Seongnam-si, Republic of Korea) was performed, and then the total RNA was extracted using a GENEzolTM reagent Total RNA Extraction Kit (Cat. Number: GZR050, Geneaid, New Taipei City, Taiwan) as indicated in the manufacturer’s datasheet. The RNA purity and concentration were then measured using a Genova Nano Micro-Spectrophotometer (JENWAY, London, UK). From the total RNA, cDNA synthesis was performed using a TOPscriptTM cDNA synthesis kit (Cat. Number: EZ0055, Enzynomics, Daejeon, Republic of Korea). To dissolve the dried pellet, vortexing was applied. Incubation was then carried out at 42 °C for 60 min, followed by heat inactivation at 95 °C for 5 min to inactivate the reaction. For real-time quantitative PCR, reactions were conducted in a volume of 10 μL using the TOPrealTM qPCR 2X PreMIX (SYBR Green with low ROX) kit (Cat. Number: RT500, Enzynomics, Republic of Korea). PCR was carried out using AZURE CIELO real-time PCR (Azure biosystems, Dublin, CA, USA), as follows: denaturation (1 cycle): 95 °C for 10 min elongation (45 cycles): 95 °C for 15 s, 52 °C for 30 s, 72 °C for 30 s, final extension: 72 °C for 7 min. The primer sequences for the cDNA used to amplify the target genes (i.e., iNOS, P53, and caspase-3) are presented in Table 1. The relative amount of gene expression was estimated according to Livak and Schmittgen [80]:

Fold change = 2−∆∆Ct

whereas:

∆∆Ct = ∆Ct reference − ∆Ct target gene
Ct = the cycle at threshold level
reference = GAPDH.

**Table 1:** Primers used for qRT-PCR in this study.

Gene	Forward Primer (5′->3′)	Reverse Primer (5′->3′)	GenBank Accession Number
iNOS	GACTGCACAGAATGTTCCAG	TGGCCAGATGTTCCTCTATT	NM_012611
P53	TAACAGTTCCTGCATGGGCGGC	AGGACAGGCACAAACACGCACC	NM_030989
Caspase-3	AGTTGGACCCACCTTGTGAG	AGTCTGCAGCTCCTCCACAT	NM_012922
GAPDH	GGTGAAGGTCGGTGT GAACG	CTCG CTCCTGGAAGATGGTG	NM_017008

### 4.10. Statistical Analysis

The data were statistically analyzed using the IBM SPSS software package version 22.0 (Armonk, NY, USA: IBM Corp). The one-way ANOVA test was used for comparison between more than two groups, and the post hoc test (Tukey HSD) was used for pairwise comparisons. Normal distribution of the data was pre-verified using the Shapiro–Wilk test and the Kolmogorov–Smirnov test. The results are presented as the mean ± standard deviation (SD), and statistical significance was set at *p* < 0.05.

## Data Availability

The data supporting the findings of this study are available in this manuscript. All the other data that support the findings of this study are available from the corresponding author upon reasonable request.

## Abbreviations

CAT: catalase; CS: ceramide synthase; GLP-1: glucagon-like peptide-1; GSH: reduced glutathione; HbA1c%: glycosylated hemoglobin; HDL-C: high-density lipoprotein cholesterol; HOMA-IR: homeostatic model assessment for insulin resistance; IL-1ß: interleukin-1beta; IL-6: interleukin-6; iNOS: inducible nitric oxide synthase; LDL-C: low-density lipoprotein cholesterol; MDA: malondialdehyde; NO: nitric oxide; p53: tumor suppressor gene; PGE-2: prostaglandin-2; RNS: reactive nitrogen species; ROS: reactive oxygen species; SOD: superoxide dismutase; STZ: streptozotocin; TC: total cholesterol; TG: triglyceride; TNF-α: tumor necrosis factor-alpha; T2DM: type 2 diabetes mellitus; TUDCA: tauroursodeoxycholic acid; VLDL-C: very low-density lipoprotein cholesterol.
